# Generation of an ICF Syndrome Model by Efficient Genome Editing of Human Induced Pluripotent Stem Cells Using the CRISPR System

**DOI:** 10.3390/ijms141019774

**Published:** 2013-09-30

**Authors:** Takuro Horii, Daiki Tamura, Sumiyo Morita, Mika Kimura, Izuho Hatada

**Affiliations:** Laboratory of Genome Science, Biosignal Genome Resource Center, Institute for Molecular and Cellular Regulation, Gunma University, Gunma 371-8512, Japan; E-Mails: horii@gunma-u.ac.jp (T.H.); t08301105@gunma-u.ac.jp (D.T.); msumiyo@gunma-u.ac.jp (S.M.); mikimura@gunma-u.ac.jp (M.K.)

**Keywords:** CRISPR, iPS, Cas9, DNMT3B, ICF syndrome, genome engineering

## Abstract

Genome manipulation of human induced pluripotent stem (iPS) cells is essential to achieve their full potential as tools for regenerative medicine. To date, however, gene targeting in human pluripotent stem cells (hPSCs) has proven to be extremely difficult. Recently, an efficient genome manipulation technology using the RNA-guided DNase Cas9, the clustered regularly interspaced short palindromic repeats (CRISPR) system, has been developed. Here we report the efficient generation of an iPS cell model for immunodeficiency, centromeric region instability, facial anomalies syndrome (ICF) syndrome using the CRISPR system. We obtained iPS cells with mutations in both alleles of DNA methyltransferase 3B (*DNMT3B*) in 63% of transfected clones. Our data suggest that the CRISPR system is highly efficient and useful for genome engineering of human iPS cells.

## 1. Introduction

Human induced pluripotent stem (iPS) cells generated by reprogramming of somatic cells with four transcription factors (Oct3/4, Klf4, Sox2, and c-Myc) have properties similar to those of human embryonic stem cells [1]. Therefore, such iPS cells are invaluable for regenerative medicine, drug screening, and biomedical research. However, to realize the full potential of human iPS cells, it will be necessary to manipulate their genomes in a precise, efficient manner. Historically, gene targeting in human pluripotent stem cells (hPSCs) has been extremely difficult [2]. Zinc-finger nucleases (ZFNs) and transcription activator-like endonucleases (TALENs) have been applied to gene manipulation of human iPS cells [3–5]. However, both ZFNs and TALENs require the design of DNA-binding proteins and complicated construction of plasmids for expression of these proteins, making these methods time-consuming and labor-intensive.

Recently, the clustered regularly interspaced short palindromic repeats (CRISPR) system, a genome engineering method using the RNA-guided DNase Cas9, has been applied to mammalian cells [6–9]. In this system, a single gene encoding the Cas9 protein and two RNAs, a mature CRISPR RNA (crRNA) and a partially complementary trans-acting RNA (tracrRNA), are sufficient for RNA-guided cleavage of foreign DNAs. Maturation of the crRNA requires RNase III and tracrRNA [10], however, this process can be bypassed by using an engineered small guide RNA (sgRNA) containing a hairpin that mimics the tracrRNA-crRNA complex and a short sequence complementary to the target DNA [11]. The Cas9 endonuclease can generate sequence-specific double-strand breaks of target DNAs bound to sgRNAs. The binding site of a target DNA must contain a protospacer-adjacent motif (PAM) (with the sequence NGG) juxtaposed to the DNA complementary region [12]. Therefore, the CRISPR RNA-guided Cas9 nuclease system requires only two molecules: the Cas9 protein and an sgRNA for host-independent gene targeting. The use of small guide RNAs for gene editing makes the CRISPR system attractive, because there is no requirement for protein design or construction of expression plasmids.

Immunodeficiency, centromeric region instability and facial anomalies syndrome (ICF) is a very rare autosomal recessive disorder characterized by facial dysmorphism, immunoglobulin deficiency, and branching of chromosomes 1, 9, and 16 after phytohemagglutinin (PHA) stimulation of lymphocytes. This syndrome is caused by mutations in DNA methyltransferase 3B (*DNMT3B*) [13–15]. The lack of DNA methyltransferase causes the hypomethylation of satellite 2 repeats, which leads to decondensation of these regions and centromeric instability, one of the hallmarks of this syndrome [15–17]. ICF syndrome is an extremely rare disorder, and only 50 cases have been reported in the world. Therefore, generation of ICF syndrome model iPS cells by gene targeting of wild-type human iPS cells would facilitate research of this rare condition. The CRISPR system has been used for targeting iPS cells recently, but the efficiency of targeting is not so high [9,18–20]. However, it is higher than the targeting efficiency of TALEN [18,20].

Here we report the efficient generation of ICF syndrome iPS cells using the CRISPR system. We obtained iPS cells with mutations in both alleles of *DNMT3B* at extremely high efficiency: 63% of transfected clones were disrupted in both *DNMT3B* alleles. Our results suggest that the CRISPR system is extremely efficient and useful for genome engineering of human iPS cells.

## 2. Results and Discussion

### 2.1. Targeting *DNMT3B* Gene in Human iPS Cells

**Figure 1:**
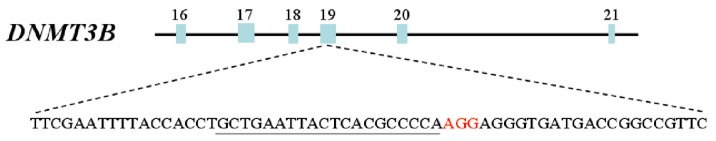
The Cas9/sgRNA-targeting site in human DNA methyltransferase 3B (*DNMT3B*). The sgRNA-targeting sequence is underlined, and the PAM (protospacer-adjacent motif) sequence is indicated in red. Exons are indicated by closed boxes.

**Figure 2:**
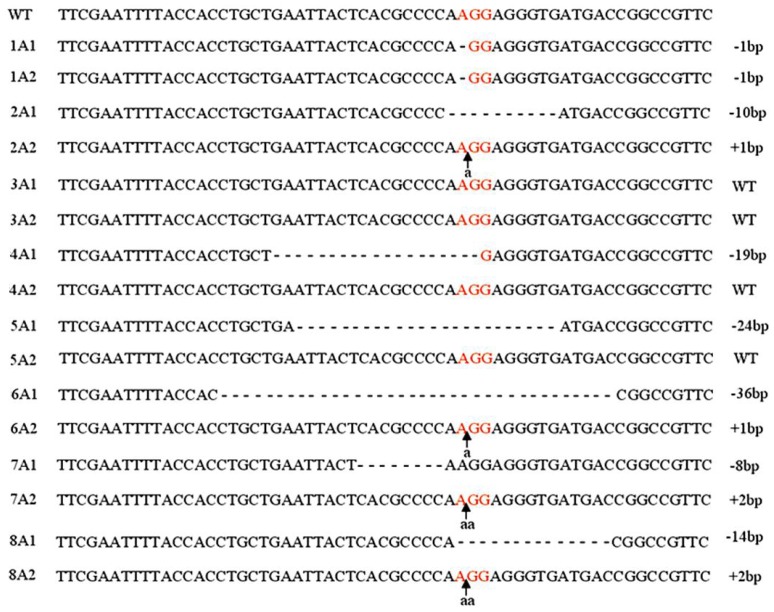
Identification of successfully targeted *DNMT3B* alleles in human iPS cells. Exon 19 of *DNMT3B* was PCR-amplified and subjected to sub-cloning and sequencing. Sequences of both alleles from a total of eight colonies are shown. The PAM sequences are shown in red, and the lowercase letters indicate insertion mutations.

A previous study of the specificity of CRISPR suggested that the DNA target site must perfectly match the PAM sequence (NGG) and the 12 bp seed sequence at the 3′ end of the sgRNA [11]; the remaining bases are believed to be less important. Therefore, we selected a 23-mer sequence (N21GG) from the target gene and used 16 bp of this sequence (N14GG) to search for homologous human genes. Sequences that did not cross-react with any other sites in the human genome were selected (Figure 1) and used in construction of sgRNA expression vectors. To maximize levels of Cas9 in the iPS cells, the expression vector was engineered to contain mammalian codon-optimized Cas9 from *S. pyogenes* under the control of a CAG promoter [9]. Human iPS cells were co-transfected with the Cas9 expression vector, an sgRNA vector targeting *DNMT3B,* and a linear puromycin marker plasmid. Transfections were performed in Primate ES Cell Medium (ReproCell Inc., Yokohama, Japan) supplemented with 10 μM ROCK inhibitor Y-27632 (Wako Pure Chemical Industries Ltd., Osaka, Japan). 12 h after transfection, the media were removed, and fresh Primate ES Cell Medium with 10 μM Y-27632 was added. Starting two days after transfection, cells were incubated transiently with 1 μg/mL puromycin for 48 h. Surviving colonies were picked seven days after the start of selection. The targeted *DNMT3B* locus was amplified by polymerase chain reaction (PCR), and the PCR products were subjected to sub-cloning and sequencing. A total of eight human iPS clones were screened (Figure 2). Seven of the eight clones (88%) were successfully targeted for at least one allele; surprisingly, six of the clones (63%) had mutations in both alleles. This efficiency is higher than those of ZFNs and TALENs, suggesting that the CRISPR system is useful for manipulating genes and constructing disease models. This high efficiency could be explained by the use of ROCK inhibitor. However, other explanation could be also possible.

Although off-target mutations were reported to be high in some cancer cell lines manipulated by CRISPR system [21,22], a recent report showed that off-target mutations in pluripotent cells and knockout mice are rare. In addition, two or more interspaced mismatches dramatically reduce Cas9 cleavage [23]. Another recent report showed that guide-RNA: Cas9 specificity extends past a 7- to 12-base-pair seed sequence. This suggests off-target mutations could be low [24].

### 2.2. Characterization of Human iPS Cells in Which *DNMT3B* Was Targeted (ICF Syndrome Model iPS Cells)

**Figure 3:**
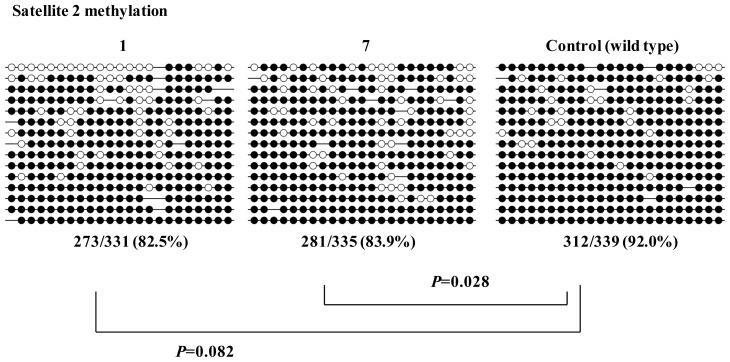
Methylation analysis of satellite 2 repeats in targeted human iPS cells. Open circles and closed circles denote unmethylated and methylated CpG sites, respectively.

Next, we evaluated the human iPS cells in which *DNMT3B* was targeted. The lack of DNMT3B methyltransferase causes hypomethylation of satellite 2 repeats, which leads to decondensation of these regions and centromeric instability, one of the hallmarks of this syndrome [15–17]. Therefore, we analyzed the DNA methylation of satellite 2 repeats using the bisulfite sequencing method. We found the satellite 2 repeats were more extensively demethylated, relative to wild-type cells, in iPS cells with targeted disruption of both *DNMT3B* alleles (Figure 3). In one clone of targeted iPS cell line 1, demethylation was not as extensive. However, because some amount of cell division is necessary for passive dimethylation to occur, serial passages of these iPS cells should result in additional demethylation. In leukocytes of ICF patients, percentages of methylation in satellite 2 are about 25% whereas those are >95% in control [16]. This extensive demethylation could be explained by the passive demethylation through some amount of cell divisions during the development. Thus, these targeted human iPS cells have characteristics of ICF syndrome, and can be used as model iPS cells for study of ICF syndrome.

## 3. Experimental Section

### 3.1. Transfection of Cells

Human pluripotent stem cells (HiPSCs) (HPS0001 201B7) [1] were cultured on mitomycin C-treated feeder cells in Primate ES Cell Medium (RCHEMD001, ReproCell Inc., Yokohama, Japan) as previously reported [1]. Before transfection, hiPSCs were resuspended using trypsin/2-({2-[bis(carboxymethyl)amino]ethyl}(carboxymethyl)amino)acetic acid (EDTA). The resuspended cells were seeded at densities of 0.8 × 10^6^ cells per well in 6-well plates, and then co-transfected with a plasmid expressing mammalian codon-optimized Cas9 under the control of a CAG promoter, a plasmid expressing sgRNAs targeting *DNMT3B* under the control of the U6 promoter, and a linear puromycin marker (Clontech, Palo Alto, CA, USA). Transfections were performed using the Lipofectamine 2000 reagent (Life Technologies, Palo Alto, CA, USA) according to the manufacturer’s instructions in Primate ES Cell Medium supplemented with 10 μM ROCK inhibitor Y-27632. 12 h after transfection, the media were removed, and fresh Primate ES Cell Medium with 10 μM Y-27632 was added. Starting 2 days after transfection, cells were incubated with 1 μg/mL puromycin for 48 h. Surviving colonies were picked 7 days after the start of selection, passaged several times, and frozen.

### 3.2. Genotyping of *DNMT3B*

To detect small modifications in the sequence of *DNMT3B* alleles, PCRs were performed using *DNMT3B*-specific primers flanking the targeted regions: 5′-CCGGCTCTTCTTCGAATTTT-3′ and 5′-CCGTGAGATGTCCCTCTTGT-3′. The PCR products were cloned into a TA-cloning vector (pCR2.1, Invitrogen, Carlsbad, CA, USA) and subjected to DNA sequencing analysis using the BigDye terminator method (ABI PRISM 3130xL, Applied Biosystems, Foster City, CA, USA).

### 3.3. Methylation Analysis of Satellite DNA

Two micrograms of genomic DNA extracted from hiPSCs was bisulfite-treated using the EpiTect Plus Bisulfite Kit (Qiagen, Hilden, Germany). One hundred nanograms of bisulfite-treated DNA was used as a template to amplify PCR products corresponding to satellite 2 (Chr. 1) using primers 5′-GAATTATTGAATAGA ATTGAATGG-3′ and 5′-TAA ATA ATA ACT CCT TTC ATT T-3′, as previously reported [25]. PCR products were gel-purified using the QIAquick Gel Extraction kit (Qiagen, Hilden, Germany) and cloned using the TOPO TA Cloning Kit (Invitrogen, Carlsbad, CA, USA). 16 positive clones for each sample were sequenced using the BigDye terminator method (ABI PRISM 3130xL, Applied Biosystems, Foster City, CA, USA). We performed Mann–Whitney *U*-test that is suitable for DNA methylation analysis of bisulfite genomic sequence.

## 4. Conclusions

A model for ICF syndrome, a very rare autosomal recessive disorder, was efficiently produced from human iPS cells using the CRISPR system. We obtained iPS cells with mutations in both *DNMT3B* alleles with extremely high efficiency (63%). These iPS cells were hypomethylated in satellite 2 repeats, which leads to decondensation of these regions and centromeric instability, one of the hallmarks of this syndrome. These findings suggest that the CRISPR system is highly efficient and useful for genome engineering of human iPS cells.
